# The Absorption of Granular Materials from the Peritoneum

**Published:** 1903-06-06

**Authors:** 


### THE ABSORPTION OF GRANULAR MATERIALS FROM THE PERITONEUM.

THE results of an interesting series of experiments which had been carried out with a view to determining the processes by which granular substances are removed from the peritoneal cavity have been recently reported by W. G. MacCallum.[1] The observations were for the most part made upon dogs, and consisted both of careful examinations of the histological structure of the peritoneum covering the under surface of the diaphragm and of the underlying lymphatic vessels, and experimental injections of substances such as carmine into the peritoneal cavity under various circumstances and noting the results. As the outcome of the first part of his inquiries he was unable to find any evidence of direct communication between the lymphatic spaces of the diaphragm and the peritoneal cavity such as have been described by V. Recklinghausen and others. Two kinds of *stomata* have been described by various histologists as connecting the peritoneal cavity with the subperitoneal lymphatics. The one kind has been supposed to consist of comparatively large openings lined by epithelial cells which are continuous with the peritoneal epithelium on the one hand, and the lymphatic epithelium on the other, the peritoneal cavity being according to this idea for practical purposes an intrinsic part of the lymphatic system—an enlarged lymphatic space. The second kind of opening has been described as intercellular, the lymphatic vessels being thought to be in direct communication with the peritoneum by means of minute intercellular spaces. MacCallum was unable to demonstrate the existence of either kind of stoma. In the second portion of his experiments in which he introduced granular material into the peritoneal cavity and observed the results both macroscopically and by means of histological sections it appeared that the leucocytes played the most important part by taking up the granules and migrating into the lymphatics with their burdens. Granules were also found in the cells lining the peritoneum, in the fixed connection tissue cells, and the epithelium of the lymphatics but not in the same quantities as in the leucocytes. Some of the granules were seen free in the lymphatics. By repeating the experiment on a dead animal whose blood had been removed by saline solution, and whose peritoneal cavity had been washed out with the same solution, it was still possible by performing respiratory movements to cause a certain amount of injection of the lymphatics. In order to eliminate any possible phagocytic action of the epithelial structures the experiment was repeated after the peritoneum had been washed with an antiseptic solution, with the same result as before, showing that neither leucocytic or epithelial activity are the sole factors. The granules were shown in these experiments to be passing through the peritoneum between and not through the cells. The respiratory movements therefore play a part, though a minor one, in the removal of granules from the peritoneum. The movements of the diaphragm by their pumping action accelerate the flow of lymph and therefore increase the rapidity of the process.

[1] Bulletin of the Johns Hopkins Hospital, May 1903.

